# Gastric polyp detection module based on improved attentional feature fusion

**DOI:** 10.1186/s12938-023-01130-x

**Published:** 2023-07-19

**Authors:** Yun Xie, Yao Yu, Mingchao Liao, Changyin Sun

**Affiliations:** 1 School of Intelligence Science and technology, University of Science and Technology Beijing, Beijing, China; 2 School of Artificial Intelligence, Anhui University, Anhui, China

**Keywords:** Gastric cancer, Deep learning, Polyp detection, Attention module, Feature fusion

## Abstract

Gastric cancer is a deadly disease and gastric polyps are at high risk of becoming cancerous. Therefore, the timely detection of gastric polyp is of great importance which can reduce the incidence of gastric cancer effectively. At present, the object detection method based on deep learning is widely used in medical images. However, as the contrast between the background and the polyps is not strong in gastroscopic image, it is difficult to distinguish various sizes of polyps from the background. In this paper, to improve the detection performance metrics of endoscopic gastric polyps, we propose an improved attentional feature fusion module. First, in order to enhance the contrast between the background and the polyps, we propose an attention module that enables the network to make full use of the target location information, it can suppress the interference of the background information and highlight the effective features. Therefore, on the basis of accurate positioning, it can focus on detecting whether the current location is the gastric polyp or background. Then, it is combined with our feature fusion module to form a new attentional feature fusion model that can mitigate the effects caused by semantic differences in the processing of feature fusion, using multi-scale fusion information to obtain more accurate attention weights and improve the detection performance of polyps of different sizes. In this work, we conduct experiments on our own dataset of gastric polyps. Experimental results show that the proposed attentional feature fusion module is better than the common feature fusion module and can improve the situation where polyps are missed or misdetected.

## Background

According to the latest 2020 Global Cancer Statistics Report published by the 2021 IARC team in an authoritative journal owned by the American Institute on Cancer Society, gastric cancer ranks 5th on the global cancer spectrum and 4th on the cause of death spectrum. According to statistics, the global number about new cases of gastric cancer in 2020 is 1089103, and the number of deaths is 768793, accounting for 5.6 and 7.7$\%$ of the total number of cases and deaths, respectively [1]. Compared with the data of recent years, the incidence of gastric cancer is also increasing. And gastric polyps, as one of the possible symptoms of gastric cancer, has also attracted more and more people’s attention. In general, gastric polyps are at risk of becoming cancerous, and the probability of risk varies for different patients. Many patients get sick without any symptoms, meanwhile, the detection of gastric polyps is mainly by gastroscopy, but this work faces two major challenges. First, the internal environment of the human stomach is very complex, the stomach structure of different patients may have huge differences, and the initial polyps are small and relatively hidden. In this way, doctors may miss the polyps when doing the endoscopy. Scientific experiments have shown that the error rate of endoscopy is as high as 26$\%$, so it is likely to lead to patients not starting to treat until the advanced stage of cancer, which has a great impact on human health [2]. Second, nowadays, there are many patients with stomach diseases, which causes a sharp increase in the data on gastric endoscopic images. The endoscopic detection technology depends on the doctor’s personal experience, this manual method is not only inaccurate and inefficient, but the final test results rely heavily on the doctor’s comprehensive ability. Moreover, even very experienced doctors are prone to miss polyps in the face of a huge workload. Once the polyps are not found in time or not treated in time, it is likely to occur lesions, so improving the detection accurate rate of stomach polyps is of great significance to people’s physical health.

To solve these problems, many traditional methods based on manual feature extraction [3–6] are applied to polyp detection. However, if the characteristics of the polyps are not obvious enough or the style of the polyps vary greatly.These methods of manually extracting features cannot learn the characteristics of the polyps well, resulting in low recognition accuracy and failure to meet the clinical requirements. The deep learning-based method has a strong feature extraction ability, which can avoid the interference of human factors and improve the accuracy of object detection, it is also used in the field of medical image processing widely. At present, deep learning methods based on convolutional neural networks have made great progress in the field of object detection. This method has two mainstream frameworks, one is two-stage detector, such as Fast R-CNN [7], Faster R-CNN [8] and Mask R-CNN [9], the other is one-stage detector, such as SSD [10], Retinanet [11], YOLO [12], YOLO9000 [13], YOLOv3 [14] and so on. Since the one-stage object detector can achieve the classification and positioning of the target at the same time, this method will be more efficient.

**Fig. 1:**
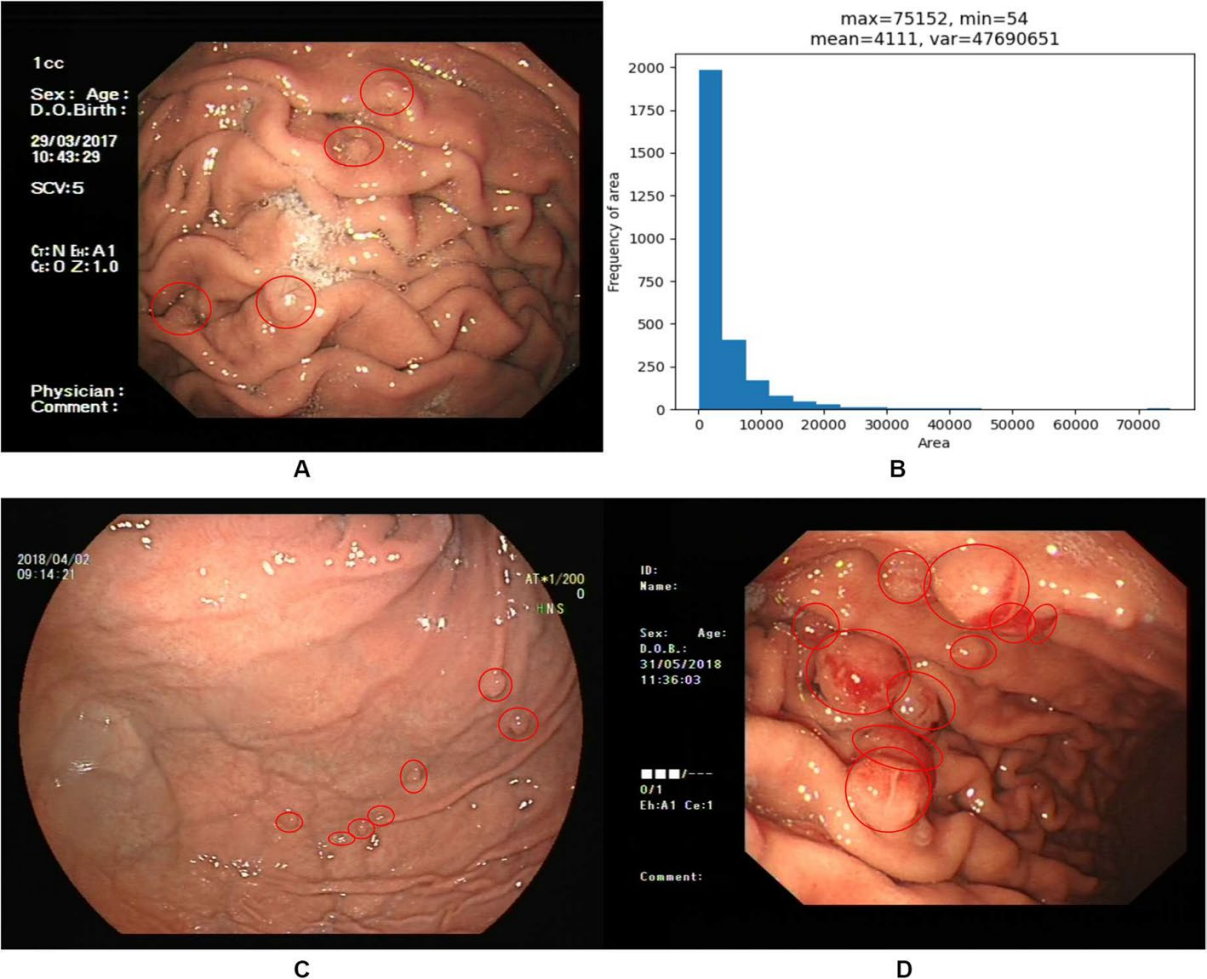
The gastric polyp about our dataset

The research of object detection in medical images based on deep learning method is of great significance to human health. Nowadays, many researchers try to study the detection of gastric polyps based on classical object detection algorithms. But most of these classical methods are used in images of natural scenes, and rarely in polyp images. At the same time, due to the differences between natural scene images and medical images in terms of the angle at which the picture was taken, the contrast between the foreground and background of the image, the distribution of targets in the image, etc. Previous target detection frameworks can be used for gastric polyp detection, but to further improve the detection of different size of gastric polyps in the complex situation, we are committed to investigate improved methods:

1. Complex environment: As shown in Fig. 1A, the stomach has a complex background environment with many gastric folds, gastric mucosa and blisters in the stomach wall, which are very similar to gastric polyps, and the contrast between the overall background of the stomach and the polyps is weak. Therefore, it is not easy to distinguish the polyps from the endoscopic images.
2. Densely distributed small polyps and polyps with great variation in shape: Fig. 1B gives the histogram of the area about polyps in our datasheet, which shows there are many small polyps; It can be seen that some polyps are small, flat, and densely distributed with the background from Fig. 1C, which can be misdiagnosed or missed easily; compare the polyps in Fig. 1D with the polyps in Fig. 1C, we can find the shape of polyps varies greatly, which makes accurate detection challenging.

Researchers have come up with lots of ways to solve the above similar problems in images of natural scenes based on the classical detectors. Lin et al. [15] added a top-down fusion path on the basic of the backbone network to fuse different scales of feature maps, it improves the detection performance of different sizes of targets. Liu et al. [16] adopted a two-way path fusion method to fuse the low-level detail information and the high-level semantic information, which helps to guarantee the integrity and diversity of features and improve the accuracy of small target detection. Hu et al. [17] used the channel attention mechanism to adjust the weights in the channel dimension of the feature map adaptively, it can make the network focus on areas that are useful for the task. Qibin et al. [18] got the long-distance dependence between the spatial location dimension and the channel dimension to locate the targets better. These methods can provide us with ideas for the detection of polyps, but cannot be used directly.

With the intention of getting more accurate feature information of polyps in the complex gastric environment, we propose an attentional feature fusion module. It can be embedded in a common CNN module to get more accurate target positioning information, by suppressing the interference from the background, we can let the network focus more attention on the areas of interest. Furthermore, the architecture of our attentional feature fusion model requires fewer multi-scale feature fusion processes to obtain more accurate attention weights, which can ensure even the network is deep, the features of polyps with different size will not be lost. In contrast to the traditional feature pyramid fusion module, our attentional feature fusion module fuses the feature maps of all layers forming the feature pyramid structure to reduce the loss of semantic information before fusion, and it combines the attention module for multi-scale feature fusion can enhance the ability to identify where and what the objects are. In our stomach polyp dataset, our attentional feature fusion module can increase each performance index by 1 to 5$\%$ compared to PAnet when embedding in a common CNN. The main contributions of our work are summarized below:

1. An improved attention mechanism is proposed, which can solve the problem that the contrast between the prospect and background of the gastric images are not strong. Our spatial attention module focuses more attention on where the target is, while channel attention module can learn what the target is better. By highlighting the important areas and paying more attention to the polyps, we can learn more discriminatory information of polyps from such an environment.
2. We propose a new feature fusion framework that forms an improved attentional feature fusion model by combining it with our attention model. It can better balance the information of different layers of the network, effectively alleviate the problem of overly focusing on adjacent feature layers and ignoring the information of other layers during information fusion, and make full use of different levels of features to obtain more accurate attention weights for the final prediction. It can also enhance the robustness of the network for small target feature extraction and improve the detection of polyps of different sizes and shapes. Our attentional feature fusion model is plug-and-play and can be used in classical detectors for better results.

The rest of this article is organized as follows. In the second section, we review the related work of CNN-based polyp detectors, attention mechanisms, and feature fusion modules briefly. In the third section, we introduce the proposed approach and the architecture of our network in detail. Section four describes the datasets required for the experiment and the details of the experiment. Section five gives the ablation study and analyzes the final experimental results. In section six, we present our conclusions and future work.

## Results

### Dataset preparation

Our experiments were conducted mainly on the home-made gastric polyp dataset. The images of gastric polyps in our dataset were taken by two different devices, the Fujinon and the Olympus. We collected images of gastric endoscopy from 406 patients within Beijing Anzhen Hospital of Capital Medical University. Of these, there were 1941 images of polyps and 329 images without gastric polyps, making a total of 2270 images. As we were performing a target detection task, we needed to know the exact location of the gastric polyps in the images, and each image was labeled by a professional doctor to ensure that there were no errors in the labeling. 1941 images containing gastric polyps were used in our task, 1433 in the training set and 508 in the validation set through random assignment. Compared with the datasets in common natural scenarios, our gastric polyp dataset contains polyps of different sizes and shapes, and the environment in which the stomach polyps are located is more complex. Most of the polyps in our dataset are distributed in various locations in the image, and some are small polyps. In addition, we also use mosaic data enhancement to combine four images into one image for training, which can obtain more image information and improve the detection performance of small objects. It also enables the model to predict polyps of different sizes on different output feature plots. Data enhancement can get richer image information to prevent the model from overfitting, and it works well on the dataset we studied.

### Implementation details

The device models used in this experiment are RTX 2080 Ti GPU and CUDA10.2, which speed up the computation of the network and thus increase the training speed of our module. We use the Pytorch development tool to conduct our experiments. All experiments were conducted on our own gastric polyp dataset. We use standard performance evaluation metrics for comparison of experimental results. During the training process, the image size is uniformly 416*416 pixels, and the initial learning rate is 0.001. The whole training process is divided into two main phases, the freezing phase and the thawing phase. In the freezing phase, the parameters of the backbone network are frozen, the parameters of the feature extraction network do not change, and the model occupies less memory. In the thawing stage, the backbone network parameters are not frozen, the feature extraction network will change, the occupied video memory is large, and all the parameters of the network will change with the training of the network. Using this training method speeds up the training of the network and prevents the trained weight parameters from being corrupted in the early stages of training. Since the main innovation of this paper is to solve the problems of gastric polyp detection in order to improve the detection effect, we implement comparison experiments on various classical backbone networks and target detection frameworks with uniform parameters to prove that our proposed attention module and feature fusion module are effective in gastric polyp detection, which can solve the problems of poor contrast of the anterior background and poor detection of small polyps in the complex environment of the stomach.

### Evaluation indicators

Mean average precision (MAP) is the most important evaluation metric for object detection tasks. The commonly used target detection index is MAP@0.5, which is the average predicted accuracy value of objects in all pictures when the IOU threshold takes the value of 0.5.

For other metrics, such as precision, recall and F1 score, we obtain the results according to the common standard of taking the IOU threshold as 0.5. Four results can be obtained by comparing the output categories of the test sample with the categories of the real labels. Among them, true positive (*TP*) means correct judgment of polyps, false positive (*FP*) means that the background is misjudged as polyps, true negative (*TN*) means that the background area is correctly judged, and false negative (*FN*) means that the polyps are misjudged as background. Precision (*P*)is expressed as the proportion of the number of polyps detected correctly (*TP*) to the number of polyps detected $(TP+FP)$. Recall (*R*) is expressed as the proportion of the number of polyps that were correctly detected (*TP*) to the number of polyps that should have been detected $(TP+FN)$. The calculation formula is as follows:

1 $${\text{Precision}} = TP/(TP + FP\;)$$

2 $$\begin{aligned}{} & {} \quad {\text {Re}}call={TP}/{(TP+FN}\;). \end{aligned}$$

F1 score is the mediated average of precision and recall. In this article, for the F1 score of single category, we can use the following formula:

3 $$\begin{aligned} F1={2PR}/{(P+R}\;). \end{aligned}$$

### Experimental results and analysis

#### Ablation experiments

In order to verify the validity of the attention module proposed in this paper, we conduct experiments on our own gastric polyp dataset, which has a more complex and challenging environment. In this part of the ablation experiment, we take the Yolov4 as the benchmarking framework, we use different structures about the combination of attention and feature fusion modules to replace the original feature fusion module for experimental comparison.

Both the position attention module and the spatial attention module are to obtain more information about the target at the spatial level, but the spatial attention module was proposed earlier in CBAM [19], which converges the maximum and average values in the channel dimension, and obtains the importance weight of the two-dimensional space, and the spatial information taken by this method is not comprehensive enough and needs to be improved. The position attention model proposed by us is inspired by spatial attention model, and for the purpose to obtain richer target position information, the average value convergence is carried out in the height dimension and width dimension, respectively, and the cross-latitude information interaction between the channel is obtained, so the importance weight of three-dimensional space is finally obtained. Therefore, it can capture not only cross-channel information, but also direction-aware and position-aware information, which can help the model locate and identify objects of interest more accurately. The experimental comparison results of the two modules also show that position attention is better than spatial attention.

The experimental comparison in Table 1 also shows that both the position attention module and the channel attention proposed in this paper are effective, and the best results can be obtained by combining the two modules. By tuning the structure of the attention module, it is known that the best combination strategy is to use the location attention module first and then pass the serial channel attention module, which can further improve the performance metrics.

**Table 1:** Ablation study on our attention module

Method	MAP@0.5 (%)	F1	*P* (%)	R (%)
Yolov4+our feature fusion model	91.52	0.87	94.94	80.07
Yolov4+our feature fusion model with spatial attention model	91.03	0.87	93.42	80.78
Yolov4+our feature fusion model with position attention model	92.17	0.89	93.65	83.99
Yolov4+our feature fusion model with channel attention model	91.05	0.87	93.44	81.14
Yolov4+our feature fusion model with first channel then position attention model	90.98	0.88	94.67	82.17
Yolov4+our attentional feature fusion model	92.33	0.89	95.92	83.63

#### Performance comparison on our datasets

To verify the usefulness of the attention module proposed in this paper, we add different attention modules to each prediction output layer for comparison with a unified backbone network and our improved feature fusion structure. Table 2 is the comparative experiment of our attention module and other classic attention modules, from which we can know that the proposed attention module can get higher precision. And for the problem that small polyps are easy to miss, the attention module proposed in this paper can improve the recall rate and reduce the omission of polyps in such a complex stomach environment.

**Table 2:** Comparison with other attention modules

Method	MAP@0.5 (%)	F1	*P* (%)	R (%)
Yolov4+our feature fusion model	91.52	0.87	94.94	80.07
Yolov4+our feature fusion model with SE [17]	92.17	0.89	93.65	83.99
Yolov4+our feature fusion model with CBAM [19]	91.05	0.87	93.44	81.14
Yolov4+our feature fusion model with SRM [20]	90.98	0.88	94.67	82.17
Yolov4+our feature fusion model with SK [21]	91.70	0.89	96.25	82.21
Yolov4+our feature fusion model with ECA [22]	92.20	0.88	95.06	82.17
Yolov4+our feature fusion model with SA [23]	91.23	0.88	93.60	83.27
Yolov4+our feature fusion model with CA [18]	91.69	0.88	93.20	82.92
Yolov4+our feature fusion model with TA [24]	91.72	0.88	94.67	82.21
Yolov4+our feature fusion model with SGE [25]	92.21	0.88	94.65	81.85
Yolov4+our feature fusion model with GAM [26]	91.71	0.88	94.63	81.49
Yolov4+our attentional feature fusion model	92.33	0.89	95.92	83.63

To demonstrate that the attentional feature fusion module proposed in this paper is effective, we compare it with other feature fusion modules. Experiments show that our attentional feature fusion framework works better than other feature fusion modules, and even than the commonly used PANet module. Table 3 shows the final comparison of results.

**Table 3:** Comparison with other feature fusion modules

Method	MAP@0.5 (%)	F1	*P* (%)	R (%)
Yolov4+ FPN [15]	90.12	0.85	92.41	77.94
Yolov4+ FPN+ASFF [27]	92.01	0.87	93.80	80.78
Yolov4+ PANet [16]	90.94	0.87	93.80	80.78
Yolov4+our feature fusion model	91.52	0.87	94.94	80.07
Yolov4+our attentional feature fusion model	92.33	0.89	95.92	83.63

As Yolov3 is a simple objection framework, for the purpose of further demonstrating the universal applicability of our attentional feature fusion model, we adopt Yolov3 network framework to perform comparative experiments of the feature fusion module, and the experimental results shown in Table 4 demonstrate the applicability of our attentional feature fusion module on various classical backbone networks. It can be found that the performance improvement of embedding our proposed attentional feature fusion module is more obvious in the use of some mainstream backbone networks, such as Shufflenetv2 [28], Mobilenetv3 [29], Ghostnet [30] and Res2Net50 [31].

**Table 4:** Verify the applicability of our attentional feature fusion module on the up-to-date backbone networks

Method	MAP@0.5 (%)	*P* (%)	R (%)
Shufflenetv2+FPN	78.70	85.90	71.40
Shufflenetv2+PANet	81.40	85.30	75.70
Shufflenetv2+our attentional feature fusion model	83.10	86.40	77.10
Mobilenetv3+FPN	76.50	79.80	69.00
Mobilenetv3+PANet	80.50	80.60	77.10
Mobilenetv3+our attentional feature fusion model	81.40	81.40	77.10
GhostBottleneck+FPN	76.30	85.40	70.00
GhostBottleneck+PANet	83.40	92.10	77.60
GhostBottleneck+our attentional feature fusion model	85.10	92.60	77.60
Res2Net50+FPN	79.50	87.20	68.60
Res2Net50+PANet	83.30	77.10	77.60
Res2Net50+our attentional feature fusion model	84.00	91.00	78.60

Our dataset is more complex than most of the datasets in nature scenes. For example, the background color of our dataset is very dark and it is similar to the polyps, and there are many folds in the stomach, all of which affect the detection of gastric polyps. We propose the attentional feature fusion module to improve the feature extraction capability and detection of small polyps in the stomach. Comparing the results of our attentional feature fusion module with those of other advanced object detectors, as shown in Table 5, we take CSP-Darknet53 as our backbone network, our attentional feature fusion module achieves a high accuracy and recall, which improves the missed detection of small polyps. The mean average precision, F1 score, precision and recall rate can reach 92.33$\%$, 0.89, 95.92$\%$ and 83.63$\%$, respectively.

**Table 5:** Summary of results for gastric polyps detection

Method	F1	*P* (%)	R (%)
CSP-Darknet53	0.76	84.00	70.00
Faster R-CNN	0.84	88.10	80.90
SSD	0.79	77.00	80.90
YOLOv3	0.81	87.80	75.70
YOLOv4	0.87	93.80	80.78
YOLOv5s	0.88	85.60	91.90
CSP-Darknet53+our attentional feature fusion module	0.89	95.92	83.63

**Fig. 2:**
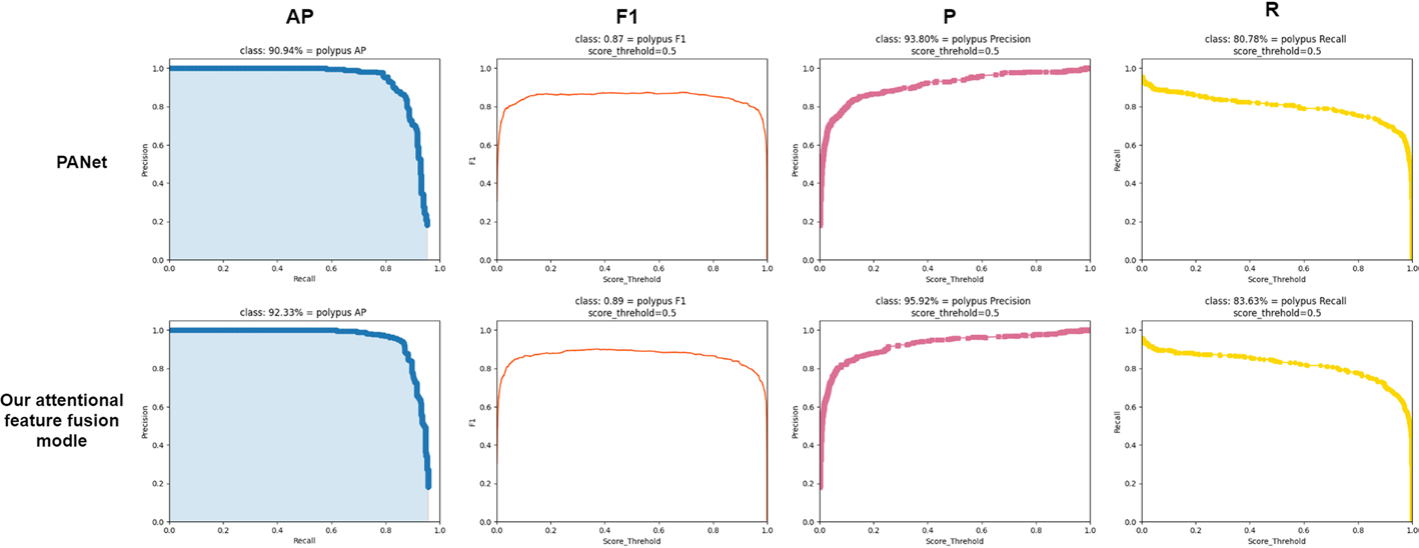
The training curve of PANet and our attentional feature fusion model

Since we mainly propose an improved feature fusion module, and the current mainstream feature fusion module is PANet. We compared the visualization results of PANet and our attentional feature fusion module on our own dataset by using the common backbone network of CSP Darknet53, as shown in Fig. 2, giving the corresponding AP curves, F1 curves, P curves, and R curves.

To demonstrate the generality and effectiveness of our proposed attentional feature fusion module on various classical target detectors, we conducted gastric polyp detection experiments using YOLOv5, YOLOv7 and YOLOv8 as benchmarks and compared the detection performance before and after the introduction of our proposed attentional feature fusion module, respectively. The results in Table 6 show that our proposed attentional feature fusion module is very effective and most of the indicators have improved.

**Table 6:** Verify the applicability of our attentional feature fusion module on the up-to-date object detectors

Method	MAP@0.5 (%)	MAP@0.5:0.95 (%)	P (%)	R (%)
YOLOv5	94.1	63.5	85.6	91.9
YOLOv5+our attentional feature fusion model	95.1	63.9	85.0	93.3
YOLOv7	90.5	59.4	89.3	83.4
YOLOv7+our attentional feature fusion model	90.7	60.1	90.9	81.4
YOLOv8	92.2	64.4	94.1	85.0
YOLOv8+our attentional feature fusion model	94.3	68.3	94.7	89.8

**Fig. 3:**
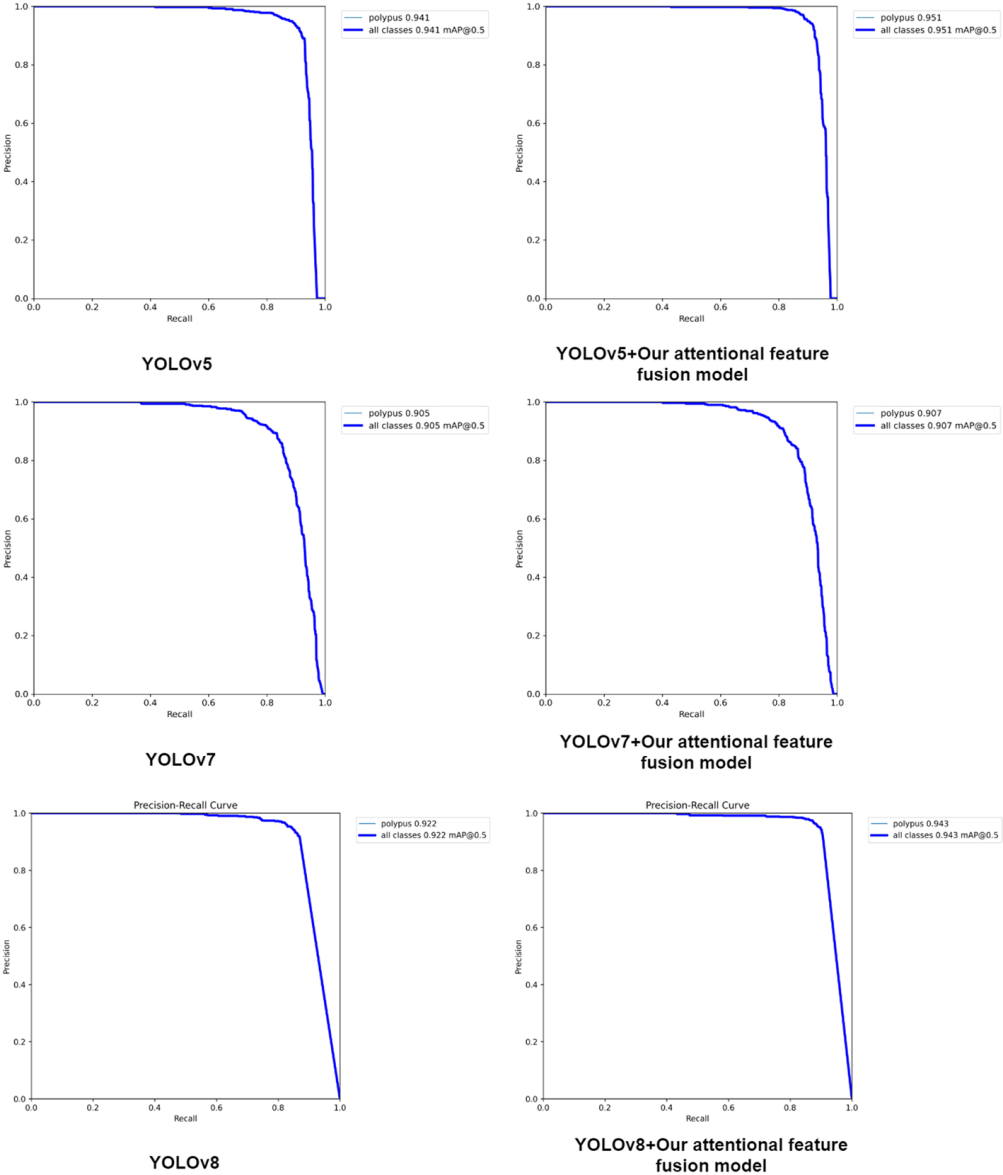
Visual PR curve comparison plot for the classic detectors

The visual comparison of the PR curves before and after the adding of our proposed attentional feature fusion module in each classical detector is given in Fig. 3. It can be found that our attentional feature fusion model has basically improved the ability of the detectors.

**Fig. 4:**
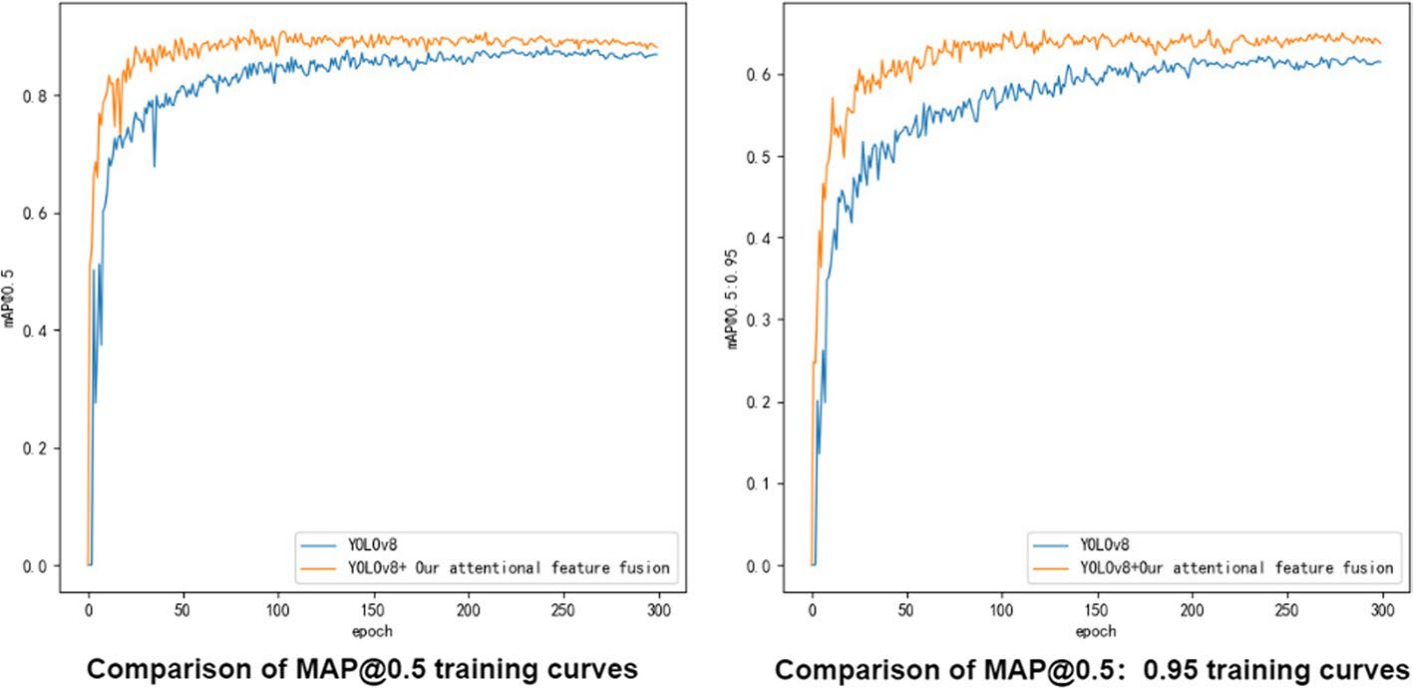
Curve comparison chart during the training process of YOLOv8

Figure 4 gives the curve comparison chart during the training process of YOLOv8, and our attentional feature fusion module is highly versatile. It can be found that compared to the original YOLOv8, the improved attentional feature fusion model is indeed more suitable for the detection of poorly differentiated gastric polyps in our complex environment.

## Discussion

### Analysis of results

Compared to other feature fusion modules, our method has higher generic performance metrics. This is mainly due to the following reasons.

**Fig. 5:**
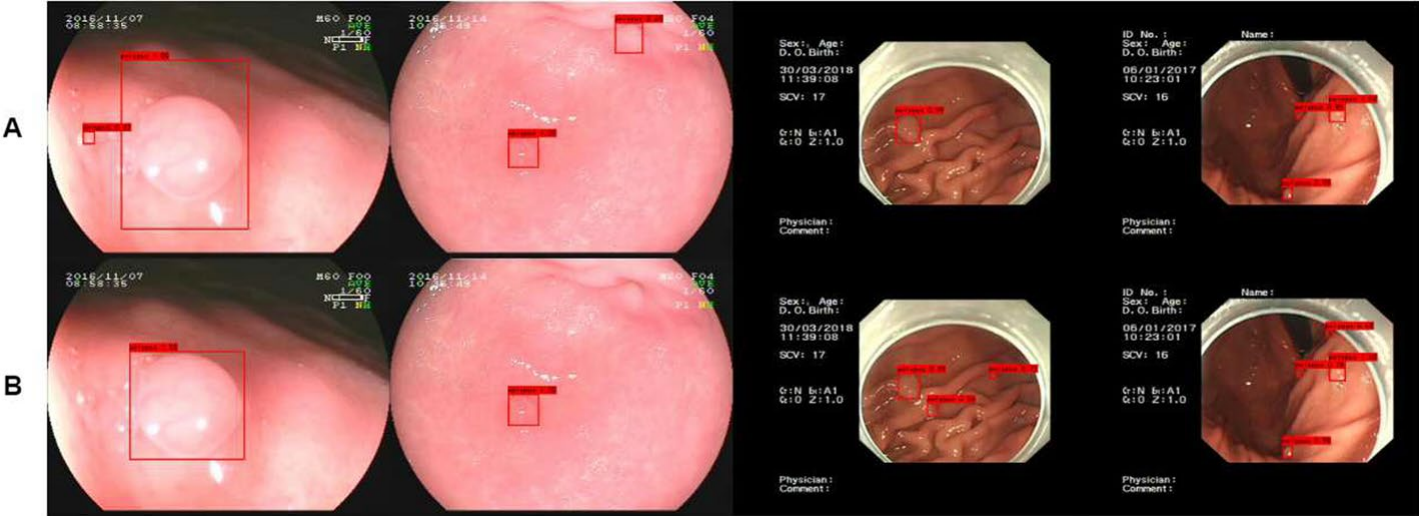
Comparison of polyp detection results on PANet and our attentional feature fusion model in the case of reflected light and stomach folds

Firstly, we propose a string-shaped position and channel attention module to mimic human vision and focus more attention on the target. For the task of detecting gastric polyps, the contrast between the polyps and the background of the stomach is not strong, for example, when obtaining an image of a gastric polyp, the reflected light is reflected on the image in the same place as the gastric polyp due to the presence of reflected light, and the folds of the stomach are very similar to the polyps, all these factors can affect the final detection results. As shown in Fig. 5A, some reflexes and gastric folds are mistaken for polyps. If we pay the same level of attention to the whole picture, it is not easy to identify polyps quickly and accurately. By using our attention module to the neck network, we can not only interact with the location information in space to capture long-range contextual information to effectively locate polyps, but also further obtain more nonlinear interaction information in the channel dimension, so that we can focus more on what the target is and where the target is during the process of training. As shown in Fig. 5B, by adding the attention mechanism, the network can better learn the characteristics of gastric polyps and reduce the probability of missed detection.

**Fig. 6:**
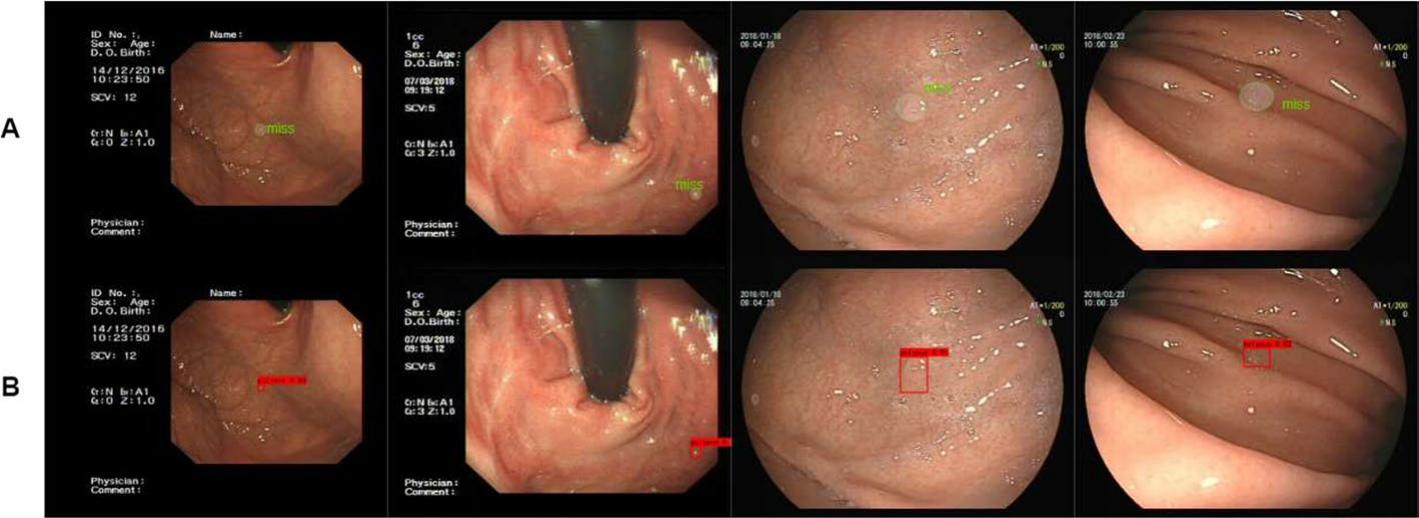
Comparison of polyp detection results on PANet and our attentional feature fusion model in the case of small polyps

Secondly, our gastric polyp dataset contains lots of small polyps, and after multiple convolution operations, the small polyps contain too few discriminative features, which leads to poor detection of our gastric polyps. Figure 6A shows the experimental results of using PAnet as feature fusion module, and we can find that the small polyps are missed significantly. Therefore, we further embed the improved attention module into the feature fusion module to form a new attentional feature fusion module, which effectively utilizes both high-level semantic features and low-level detail features for multi-scale prediction and improves the ability to extract small polyp features. As shown in Fig. 6B, small polyps are accurately detected, and our attentional feature fusion module can indeed improve the detection effect of small polyps by experimental comparison.

### Network visualization with Grad-CAM

**Fig. 7:**
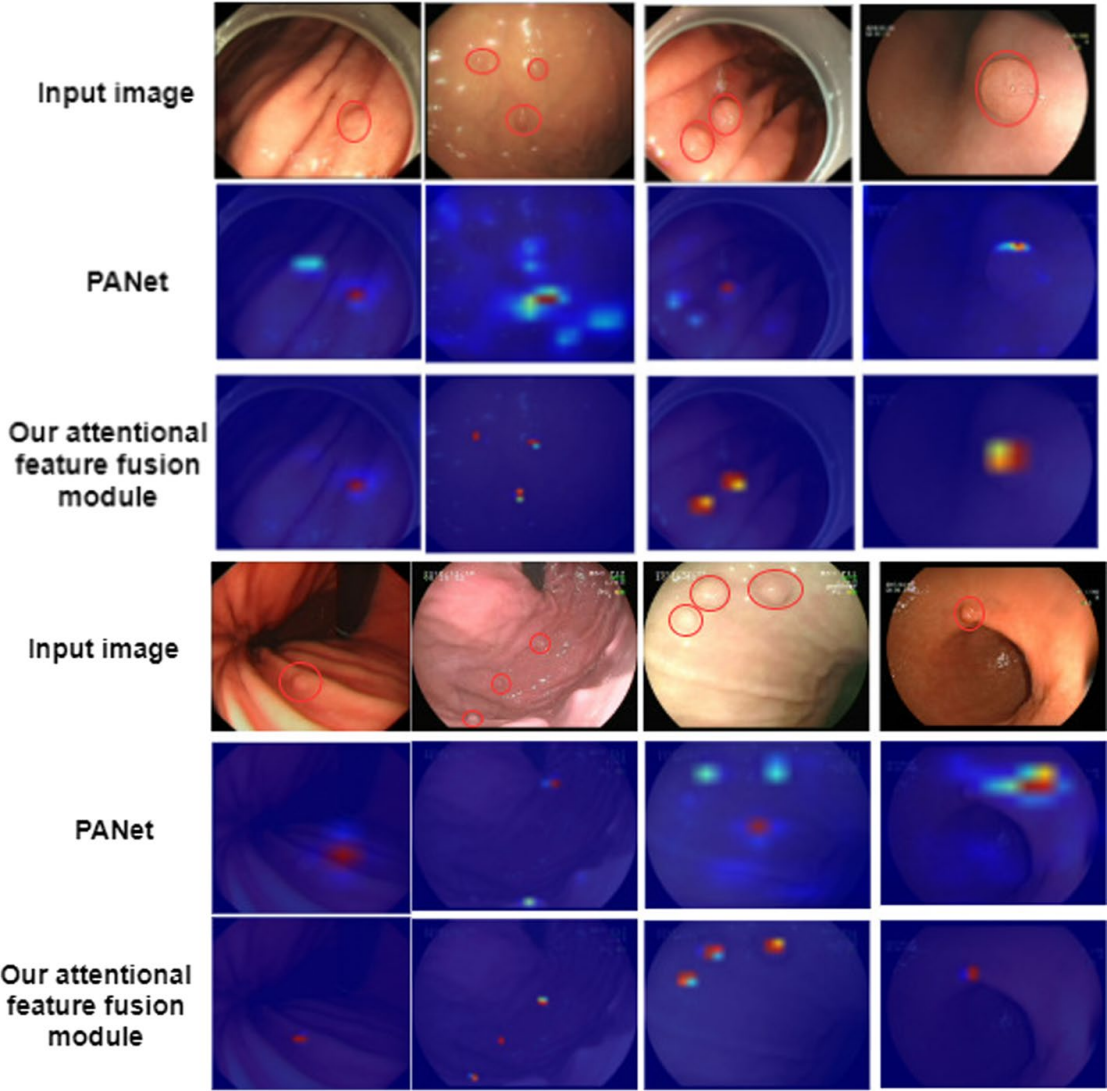
Visualization of Grad-CAM result

We hypothesize that the interaction of position information and channel information provided by the position and channel attention modules is helpful for our network to learn more meaningful features of gastric polyps. Since neural networks are usually considered as black boxes in which the principles are not well explained, we do not know why the network makes such predictions or where it focuses, so to test the proposed hypothesis, we provide the visual pictures of the Grad-CAM [32] sample through which we can draw the heat maps as shown in Fig. 7. In this way, we can know, corresponding to a given class, which regions in the network are attended to. The gradient information from the last convolutional layer of the convolutional neural network is used by Grad-CAM to assign significant values to each neuron for a specific attention decision, which can be used to interpret the activation in any layer of the deep network. As shown in Fig. 13, our method can capture more accurate polyp boundaries from our self-made data set and the previous method of detecting small polyps is easy to miss polyps because the background color is not strong. In some scenarios, when the attentional feature fusion module proposed by us is used on the benchmark model, polyps that cannot be correctly identified by the baseline model can be identified, and the probability of false detection of polyps can also be reduced. The heat map provided can support our understanding of the intrinsic role of the attentional feature fusion module, which can capture richer and more discerning contextual information for specific target category detections. Compared with the benchmark model, the feature of the attentional feature fusion module proposed in this paper is meaningful, and it is more helpful to improve the detection of small targets.

## Conclusion

In this paper, we propose a new attentional feature fusion module. In this work, our proposed attention mechanism can focus on where and what the target is, and is applicable to detect polyps in stomach images with low contrast between foreground and background. Further, the improved attention module is embedded in our feature fusion module to form a new attentional feature fusion module, which can use the high-level semantic features and low-level detail features for fusion to obtain more accurate attention weights for the final multi-scale prediction. Through experimental comparison, our attentional feature fusion module can indeed improve the object detection index of polyps. Since there are more and more studies on the attention mechanism, and the fusion of feature maps of different layer sizes using the attention module may yield unexpected results, in the future, we plan to further improve the attention module and investigate effective fusion methods for feature maps of different sizes.

## Related work

### CNN-based gastric polyp image detectors

As there is no public gastric polyp dataset, so the research of gastric polyp is not much. The present research is start with self-made datasets. Our paper is also by using our own gastric dataset to implement our experiments. Here, we are going to have a view about the past researches on gastric polyp.

About the CNN-based colon polyps detection methods, [33] used ROI alignment operation, GIoU loss function and soft NMS method on Faster R-CNN to get better gastric polyp detection results. [34] designed the convolutional neural network architecture and used data augmentation and weight attenuation techniques to train the convolutional neural network model to solve the problem of the different sensitivity of different types of endoscopic images on detection. Based on different backbone networks, a computer-aided diagnosis system based on deep learning is established in [35] to automatically detect gastric polyps. [36] designed an algorithm that uses plaque image results to locate polyp areas, helping endoscopists find polyps during real-time endoscopy. For polyps that vary in size, shape and texture, [37] based on Yolov3 to improve the detection of gastric polyps. There are also some other deep learning-based polyp detection methods, such as optimized anchor generation method [38], image filtering method [39], fusion and fine-tuning method [40] and image enhancement method [41].

**Fig. 8:**
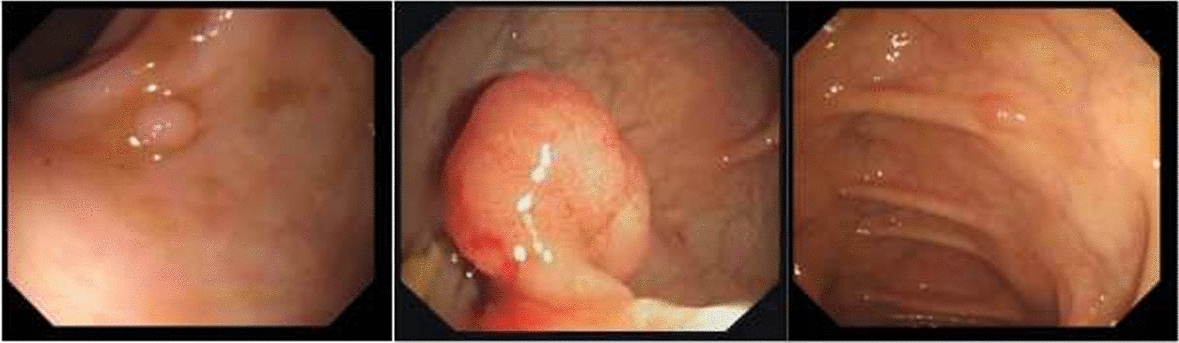
The colonic polyp images in public dataset

The previous method was either based on the improvement of gastric polyp detection based on the relatively advanced network at that time, or the improvement of detection according to the characteristics of the dataset. However, as most of the gastric polyp images we used shown in Fig. 8 are significantly smaller and denser than others, and the stomach environment in our research is complex and not distributed in the center of the images like other people’s datasets. Therefore, it is still a challenging thing to detect the gastric polyps accurately, and our research focused on improving the detection of polyps in scenes with low background contrast and dense scenes. Therefore, we need to further study the new object detection methods based on the characteristics of our gastric polyp datasets.

### Attention machine

In the past, many algorithms had studied the use of attention mechanism in backbone network to enhance the feature extraction capabilities of the network, thereby improving the performance of image classification and object detection tasks. The most common methods are channel attention and spatial attention. The classical method is SENet [17], which used global average pooling operation to get the importance weight of each channel dimension. However, this method only captures the correlation of the channel dimension and ignores the information interaction of the spatial dimension. Subsequently, the combination of global average pooling operation and global maximum pooling operation has been proposed in [19]. Moreover, this method adopts parallel channel attention and spatial attention, which can capture the importance of the channel dimension and learn the weights of attention in the spatial dimension. Since the interaction is only in channel dimension or two-dimensional spatial dimensions, the interaction of the three-dimensional space is not well captured. It is easy to lose the positional information which is useful for target detection, so the position attention module [18] was proposed, it can capture long-range dependencies along one spatial direction and retain precise location information along another spatial direction. However, these methods ignore the interaction of three-dimensional information, so a new method [24] was proposed to get the attention weight using the three-branch structure to capture cross-dimensional interactions, but this approach requires a large floating-point operation. Our attention module is motivated by the CBAM, but unlike above approaches, our aim is to learn information about the location of polyps in complex scenes, so that we can further go to discriminate whether polyps are present at the current location. Besides, we adopt the soft pooling which is not used in attention module before, it can adjust the importance of each feature map by softmax weights.

### Feature fusion

**Fig. 9:**
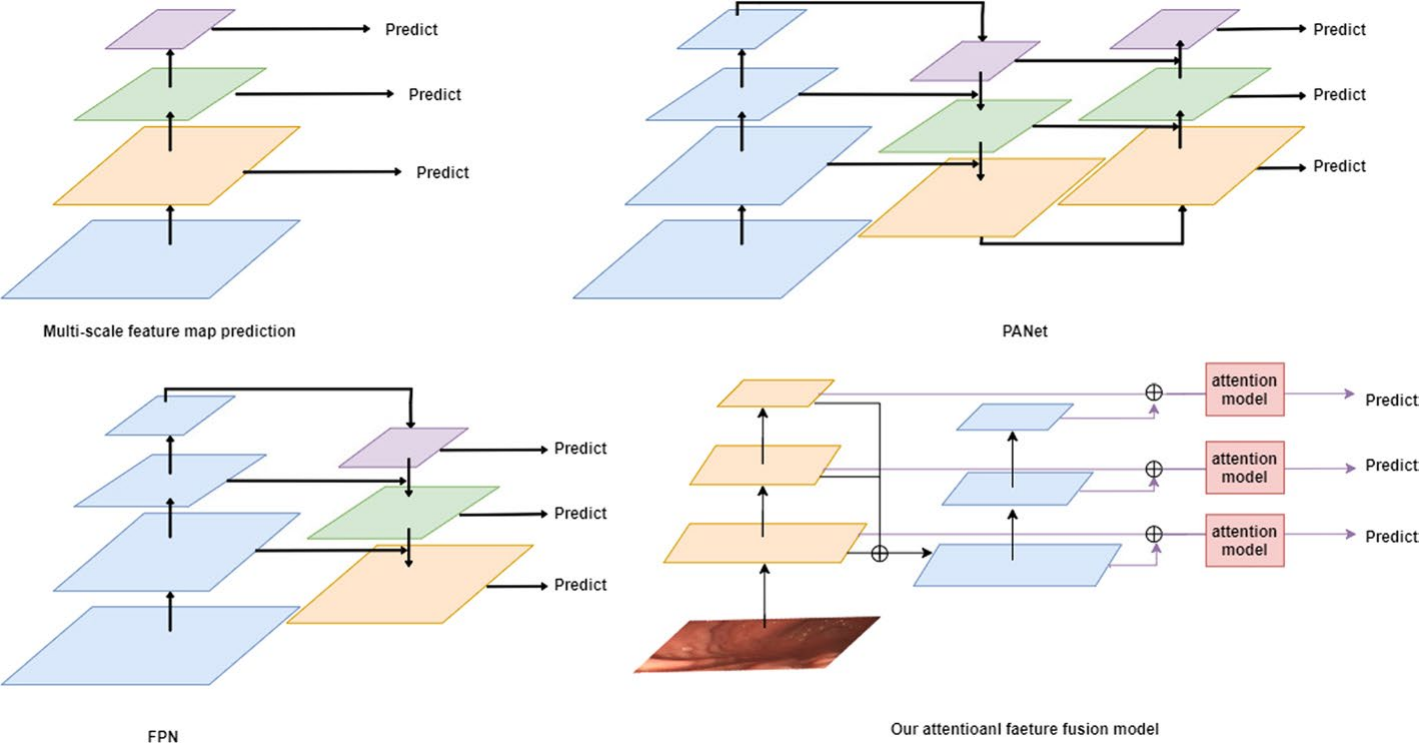
The structure of our attentional feature fusion model and other classical feature fusion models

Although the existing object detection algorithms perform well in natural scenes, the effect of these methods for gastric polyps especially small polyps is still not good enough. The small targets are very small in the original feature map, after multiple convolutional operation, the extracted details about the small targets are little, which leads to the result that some small targets can be missed. Feature fusion is one of the ways to solve this problem. Liu et al. [27] proposed an adaptive spatial feature fusion method, allowing the network to learn the spatial weights of different sizes of feature map adaptively, and then integrate them by coefficient weighting. Tan et al. [42] adapted a weighted two-way feature pyramid network that simply used scalars for weighting, which achieves a more efficient multi-scale fusion method to balance the feature information of different scales. Liu et al. [16] added a bottom-up path on the basic of the FPN, which shortens the distance from the shallowest large-scale feature map to the final small-scale feature map for detection. Previous method adjusts the number of channels to fuse the information of two different size of feature maps by convolutional operation with a convolutional kernel of one, which obviously will lose some information. Our feature fusion module fuses the last three layers of feature maps of the backbone network directly, and then further forms a spatial pyramid structure, which can reduce the semantic information loss caused by the reduced number of channels, and at the same time, through the attention module, makes the features of the final predict output layer more discriminative. Our feature fusion module fuses the last three layers of feature maps about the backbone network by concatenate in channel dimension, which contains richer information relative to the polyps. And then further forms a spatial pyramid structure, for the purpose to output the richer feature map at the corresponding scale through a bottom-up fusion path, which can reduce the semantic information loss caused by the reduced number of channels and alleviate the feature gap during the fusion process. At the same time, through the attention module, makes the features of the final predict output layer more discriminative. The comparison of the structure about our attentional feature fusion model and other classical feature fusion models is shown in Fig. 9.

## Methods

**Fig. 10:**
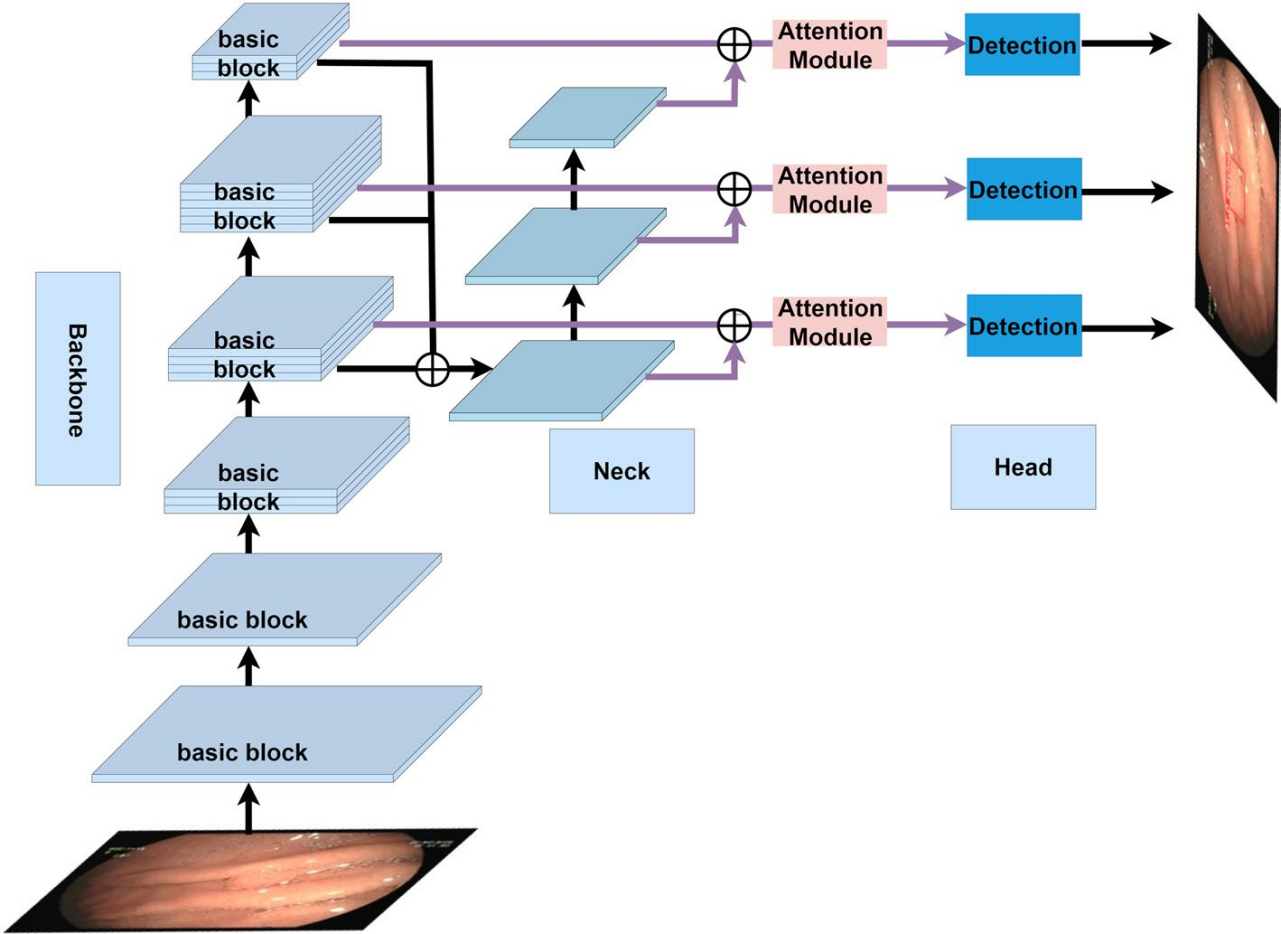
The architecture of our attentional feature fusion model-based detector

In this section, we mainly introduce the details of our attentional feature fusion module. The overall architecture of our attentional feature fusion module-based detector is shown in Fig. 10. We design and introduce the attention module to enhance the module’s ability to detect the objects from the complex background. Our attentional feature fusion module can get more semantic information and alleviate the huge gap between the fusion of different scale of feature maps. The new attentional feature fusion method effectively enriches the semantic information and improves the discrimination of the extracted features, which can be useful for our gastric polyp detection, especially for small polyps. Below, we will take a closer look at the attention module and the improved attentional feature fusion module.

### Position and channel attention machine

**Fig. 11:**
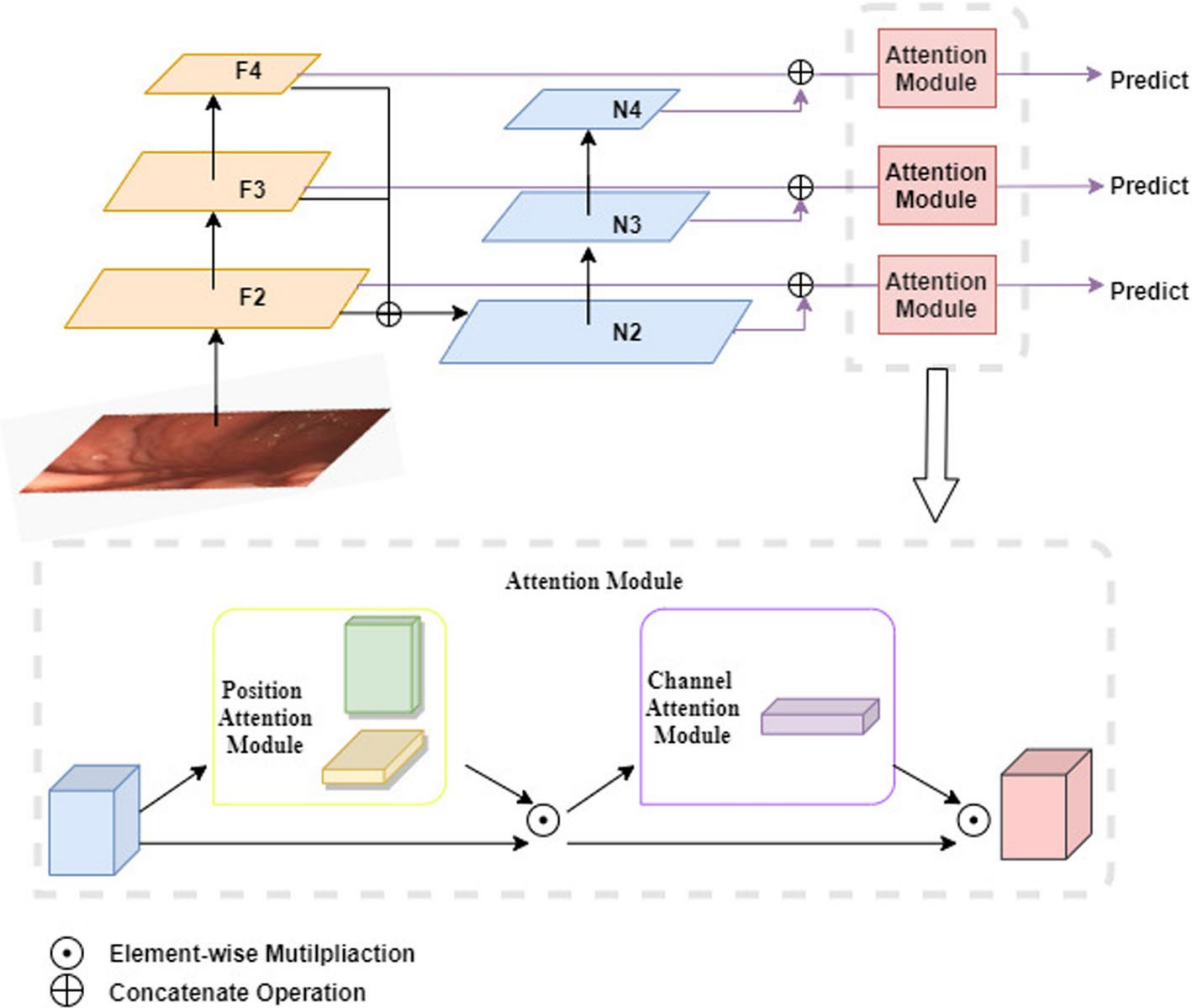
An overview of the proposed attentional feature fusion model

There are many stomach folds in the gastric endoscope picture, it is easy to confuse them with the polyps. And the color of polyps and the background is very similar, which will make it difficult to find the polyps. To solve the problem, we propose a kind of attention module used in our attentional feature fusion model. As shown in Fig. 11, the parallel position attention and channel attention module are used, it can focus on what and where the object are. The position attention module enhances the feature expression of the important areas to capture the important location information containing the target, then the channel attention module is used to encode the characteristics of the spatial dimension to further discern whether the targets are included in the region of interest.

#### Position attention module

**Fig. 12:**
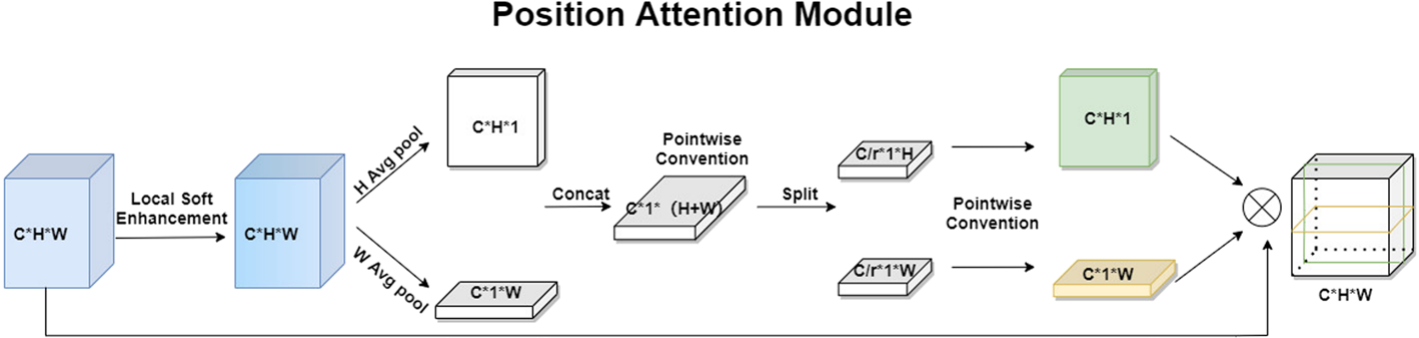
Position attention module

In the past, most spatial attention modules obtained two-dimensional spatial attention maps by global averaging pooling operations for emphasizing meaningful features in spatial extent, but spatial attention modules ignore direct information interaction with channel dimensions. Based on CANet [18], we propose a position attention module as shown in Fig. 12. Considering that in the gastroscopic image, the feature information around the polyp is benefit for the detection task, we first perform a local soft enhancement operation on the input feature map to enhance the useful information in the image, and then perform an average pooling operation on the height and width dimensions of the feature map, respectively, which captures the interaction between the spatial dimension and the channel dimension.

In the position attention model, it is of great importance to obtain the spatial structure of the target, as each branch can capture the long-distance dependence of the input feature map along one of the spatial directions. For the purpose of integrating the spatial coordinate information more efficiently and studying the nonlinear interaction of the two spatial dimensions, we use the concat operation to stitch and fuse the feature map, and then integrate the important information of the two spatial directions by pointwise convolution, which can help to obtain more complementary information related to the location. Furthermore, the split operation is used to weight the attention weights of the two directions back to the corresponding directions of the original feature map. This not only integrates the interaction of three-dimensional space, but also learns the long-distance dependencies that contain the location information of objects. Finally, by using the hard-Swish activation function, we can get the position attention feature map. The specific process for getting position attention map is as follows:

For the input feature map$X'$, a local soft enhancement operation is carried out to adjust the spatial information within a certain range. Specifically, this operation is like the convolution operation with a convolution kernel size of two. The parameters of the convolutional kernel ${{w}_{i}}$ are not learned by the process of training, but the Softmax weights, which are obtained by the four values in each spatial range of two by two, and the adjusted feature map is expressed as follows:

4 $$\begin{aligned}{} & {} {{w}_{ji}}=\frac{{{e}^{{{x}_{ji}}}}}{\sum \limits _{i=1}^{4}{{{e}^{{{x}_{ji}}}}}}, \end{aligned}$$

5 $$\begin{aligned}{} & {} \quad {{X}_{j}}={{w}_{i}}*X_{_{j}}^{i}=\sum \limits _{i=1}^{4}{{{w}_{ji}}{{x}_{ji}}}, \end{aligned}$$

where ${{x}_{ji}}$ represents the four pixel values corresponding to the *jth* position of $X'$,${{w}_{ji}}$ represents the soft-active weight value for pixel values of the four positions.${{X}_{j}}$ is the pixel value at the *jth* position of$X'$ after local soft enhancement operation.

Secondly, to capture the long-range interactions spatially with precise location information, the operation of global average pooling is conducted in both height and width dimension of the feature map, and the formula is as follows:

6 $$\begin{aligned}{} & {} X_{c}^{h}(h)=\frac{1}{W}\sum \limits _{i=1}^{W}{{{x}_{c}}}\left( h,i \right) , \end{aligned}$$

7 $$\begin{aligned}{} & {} \quad X_{c}^{w}(w)=\frac{1}{H}\sum \limits _{i=1}^{H}{{{x}_{c}}}\left( j,w \right) , \end{aligned}$$

where $$X_{c}^{h}(h)\in {{R}^{C\times H\times 1}}$$,$$X_{c}^{w}(w)\in {{R}^{C\times 1\times W}}$$.

Then, the feature maps are reordered by the permute function and the global representation of the two feature maps is connected through a concatenate operation. Further, through the pointwise convolution operation, the module can get nonlinear interaction information between height and width dimension. We use hard-Swish activate function to get the attention maps of different spatial dimensions. Finally, the attention maps are multiplied in corresponding spatial dimensions of the original feature map. The formula is expressed below:

8 $$\begin{aligned}{} & {} {{X}_{1}}=Cat([permute(X_{c}^{h}(h)),X_{c}^{w}(w)]), \end{aligned}$$

9 $$\begin{aligned}{} & {} \quad {{F}_{1}}={{f}^{C}}(split({{f}^{C/r}}({{X}_{1}}),[H,W])), \end{aligned}$$

10 $$\begin{aligned}{} & {} \quad {{w}^{h}}=h\_Swish(F_{1}^{h}), \end{aligned}$$

11 $$\begin{aligned}{} & {} \quad {{w}^{w}}=h\_Swish(F_{1}^{w}), \end{aligned}$$

12 $$\begin{aligned}{} & {} \quad {{U}_{c}}(i,j)=X_{c}^{'}(i,j)\times w_{c}^{h}(i)\times w_{c}^{w}(j). \end{aligned}$$

Among them, *permute* represents the adjustment operation in the height and width directions of the feature map, represents the point-by-point convolution operation with a convolutional kernels size of *k*, and *split* represents the splitting operation in the width dimension of the feature map.

#### Channel attention module

**Fig. 13:**
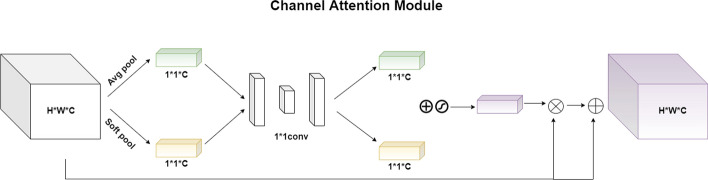
Channel attention module

Global average pooling operation uses the average value to describe each channel of the feature map, so the extracted feature information is not much different, while the global maximum pooling operation can extract the feature of the most important location. However, these operations often lose some useful information. Our channel attention mechanism uses both global soft pooling operation and global average pooling operations. Among them, soft pooling is a fast and efficient index-weighted activation method that can retain more information, the weight value of each channel obtained will be more representative and precise, so it is very suitable for the task scenarios where the contrast between the target and the background is not obvious. Compared to methods based on maximum and average values, the use of soft activation function can produce normalization results, and the distribution of weight is proportional to the activation value for each location on the channel dimension relative to the activation value for all locations. Soft pooling is a more precise method, as all the positions within each channel contribute to the final output and high activation is more dominant than low activation, it can balance the effects of average pooling and maximum pooling while also taking full advantage of the effective characteristics. The channel attention mechanism proposed in this paper is shown in Fig. 13, and the specific function process is as follows:

Firstly, the input feature map is $U\in {{R}^{C\times H\times W}}$,through two parallel branches, global average pooling and global soft pooling, respectively. The two-dimensional real values of each channel are compressed into a numerical value. Since each real number is calculated according to the two-dimensional eigenvalue, the extracted feature is equivalent to having a global sensing field, and the number of channels remains unchanged. Finally, two feature maps of size $C\times 1\times 1$, ${{s}_{1}}(c)$ and ${{s}_{2}}(c)$, are obtained. The formula is expressed as:

13 $$\begin{aligned}{} & {} {{s}_{1}}(c)={{F}_{ga}}({{U}_{c}})=\frac{1}{H*W}\sum \limits _{i=1}^{H}{\sum \limits _{j=1}^{W}{{{U}_{c}}}}\left( i,j \right) ,{{s}_{1}}\in {{R}^{C}}, \end{aligned}$$

14 $$\begin{aligned}{} & {} \quad {{w}_{c}}(i,j)=\frac{{{e}^{{{U}_{c}}(i,j)}}}{\sum \limits _{i=1}^{H}{\sum \limits _{j=1}^{W}{{{e}^{{{U}_{c}}(i,j)}}{{U}_{c}}}}\left( i,j \right) }, \end{aligned}$$

15 $$\begin{aligned}{} & {} \quad {{s}_{2}}(c)={{F}_{gs}}({{U}_{c}})=\sum \limits _{i=1}^{H}{\sum \limits _{j=1}^{W}{{{w}_{c}}(i,j)*{{U}_{c}}}}\left( i,j \right) ,{{s}_{2}}\in {{R}^{C}}. \end{aligned}$$

Secondly, the parameters obtained after pooling are used as the weight values of each feature map in the channel dimension, and then through two convolution operations, the channel is first reduced in dimensionality and then upgraded in that dimension, thus capture the information interaction cross-channel. It is more lightweight and efficient than the previous method of using a fully connected layer.

Then, the two normalized channel weight maps obtained are fused by an addition operation, and the weight values between 0 and 1 are obtained by the activation function. Finally, multiplying the weight values by each channel value of the original input feature map so that channel attention can be embedded without changing the size of the original feature map. The attention mechanism of this article is lightweight and effective, what’s more, it can be easily embedded in the classic backbone network.

### Attentional feature fusion module

At present, many algorithms consider the method of feature fusion to improve the detection performance of multi-scale targets. The commonly used network is still PANet, and the subsequent YOLO series networks mainly adopt this feature fusion structure. It adopts a top-down path and a bottom-up path to fuse feature maps of different sizes, thereby improving the performance of object detection. However, in this model, the low-level feature map lacks the guidance of the highest layer feature map, so the fusion of information for the final detection layer is still insufficient. Meanwhile, our polyp dataset contains many small polyps, some of which are relatively dense, and some are very similar to the folds of the stomach, which makes it easy to leak polyps in a complex environment. We need to design a new structure which can be applicable to our datasets. In this paper, we propose a new attentional feature fusion module; the structure block diagram is shown in Fig. 5. We use the proposed attention module the improved feature fusion module to form our own attentional feature fusion module.

Firstly, we use the nearest neighbor interpolation and concatenate operations to fuse the features in the backbone network of last three layers (from F2 to F4). Compared with other fusion methods, it is easier to fuse features of different scales by concatenation in the channel dimension, and as the number of features (number of channels) increases, the obtained features are more abundant. Secondly, we will fuse the features through a series of convolutional operations, which can further extract the deep semantic information. Then, due to the large semantic gap between the feature maps of different scales, we further integrated the three-layer multi-scale feature map (from N2 to N4) with the feature map of the last three layers in the backbone network through the convolutional operation and the stitching operation in the channel dimension. The multi-scale feature maps of the input in the last three layers are fused with the feature map of the previous layer, and the feature map of the output is used as the input to the next layer of feature map and the prediction of the target. In this way, the final output feature map contains more information than the feature map of the original backbone network, and the features are expressed more comprehensive. For the purpose of copping with the complex gastrointestinal environment in our dataset, we add a corresponding attention module before each output prediction layer to capture more information related to the target location, and obtain the entirely accurate boundary information of the target.

## Data Availability

Not applicable.
